# Attitudes Towards Treatment as Prevention Among PrEP-Experienced Gay and Bisexual Men in Australia

**DOI:** 10.1007/s10461-023-04019-x

**Published:** 2023-03-06

**Authors:** Alexander Dowell-Day, Timothy Dobbins, Curtis Chan, Doug Fraser, Martin Holt, Stefanie J. Vaccher, Brent Clifton, Iryna Zablotska, Andrew Grulich, Benjamin R. Bavinton

**Affiliations:** 1 School of Population Health, Faculty of Medicine and Health, UNSW Sydney, Sydney, NSW Australia; 2 The Kirby Institute, UNSW Sydney, Sydney, NSW Australia; 3 Centre for Social Research in Health, UNSW Sydney, Sydney, Australia; 4 National Association of People with HIV Australia, Newtown, NSW Australia; 5 Westmead Clinical School, Faculty of Medicine and Health, University of Sydney, Westmead, Australia; 6 Sydney Infectious Diseases Institute, University of Sydney, Westmead, Australia; 7 Western Sydney Sexual Health Centre, Western Sydney Local Health District, Parramatta, Australia

**Keywords:** Gay and bisexual men, Men who have sex with men, Pre-exposure prophylaxis, Treatment as prevention, HIV

## Abstract

The introduction of HIV pre-exposure prophylaxis (PrEP) has the potential to impact the attitudes gay and bisexual men (GBM) who consequently choose to take PrEP have towards treatment as prevention (TasP), and the extent to which they are willing to have condomless anal intercourse (CLAI) with an HIV-positive sexual partner who has an undetectable viral load (UVL). Using a cross-sectional sample from an observational cohort study conducted from August 2018 to March 2020, we examined the extent to which PrEP-experienced GBM are willing to have CLAI with a partner who has a UVL. Simple and multiple logistic regression models were used to identify associated variables. Of the 1386 participants included in the analyses, 79.0% believed in the effectiveness of TasP, and 55.3% were willing to have CLAI with a partner who has a UVL. Wiling participants were less worried about getting HIV when taking PrEP and more likely to believe in TasP. Further research is needed to better understand the gap between belief in TasP and willingness to have CLAI with a partner who has a UVL among PrEP-experienced GBM.

**Supplementary Information:**

The online version contains supplementary material available at 10.1007/s10461-023-04019-x.

## Introduction

In the last decade, reductions in HIV transmission among gay and bisexual men (GBM) and other men who have sex with men in some countries can be attributed to the increased use of biomedical HIV prevention strategies such as treatment as prevention (TasP) and HIV pre-exposure prophylaxis (PrEP) [1]. Both TasP and PrEP have been proven to prevent HIV transmission [2–4], and HIV diagnoses among GBM in multiple high-income countries has steadily declined since the mid-2010s [5–7]. The emergence of TasP and PrEP changed the range of sexual practices that can be considered ‘safe sex’ for HIV, a particularly important change for GBM, many of whom dislike condoms and consider them a barrier to pleasure and intimacy [8, 9]. PrEP provides HIV-negative individuals with the agency to rely on an HIV prevention strategy they can use independently of a sexual partner [9]. In contrast, other HIV prevention strategies necessitate preventing transmission by proxy through reliance on a partner’s HIV prevention strategy (e.g. serosorting) [10], or reliance on condoms, the use of which is contingent on agreement with a sexual partner. PrEP-experienced GBM may have a higher belief in TasP, and a greater willingness to have condomless anal intercourse (CLAI) with an HIV-positive sexual partner who has an undetectable viral load (UVL), due to their experience with taking PrEP. This is significant, as such increases may improve GBM’s confidence when relying on TasP, and indicate participants’ increased comfort with having CLAI with HIV-positive partners with a UVL.

Early TasP-focused research found belief in the effectiveness of TasP as a population-level approach to HIV prevention was very low among Australian GBM, at 2.6%, in 2013 [11], increasing to 13.1% by 2015 [12]. At that time, the effectiveness of TasP had only been demonstrated in heterosexuals [13], and knowledge of evidence for TasP among GBM was low [14]. The effectiveness of TasP in GBM was demonstrated in two international cohort studies, reporting their results from 2016 [2, 4]. In 2016, the global Undetectable = Untransmittable (U = U) campaign was launched to increase awareness of TasP and reduce HIV stigma [15]. The campaign has been endorsed by organisations in over 100 countries, with national and sub-national U = U campaigns being established [12, 15]. With new evidence and increased education, GBM’s belief in the effectiveness of TasP continued to increase. One national study from Australia which included HIV-negative and HIV-positive GBM found 34.6% reported a belief in TasP’s effectiveness in 2019 [12]. A similar increase was seen in the United States among GBM in Atlanta from 1997 to 2015 [16]. By 2018, 53.2% of GBM in the United States reported that they believed in U = U [17]. In both Australia and the United States, PrEP-experienced GBM have been found to be more likely to believe in the effectiveness of TasP as an HIV prevention strategy, compared to other HIV negative GBM not on PrEP [17, 18]. These findings highlight increasing levels of belief in TasP among GBM, and the differences in attitudes towards TasP held by PrEP-experienced and PrEP-naive GBM.

Though belief in the effectiveness of TasP is increasing among GBM, they tend to distrust TasP on a personal level. In 2012, 20.5% of GBM, and just 11.1% of HIV-negative or untested GBM, reported they were willing to have serodiscordant CLAI with an HIV-positive partner taking HIV treatment [14]. A 2016 state-based study from Victoria found 6.0% of HIV-negative or untested men not on PrEP were comfortable having CLAI with a HIV-positive partner with an undetectable viral load [18]. Previous research on PrEP-experienced GBM’s attitudes to TasP has focused on belief in effectiveness of TasP to prevent HIV transmission, rather than willingness to have CLAI with a partner who has a UVL [12, 17]. However, one study found HIV-negative GBM on PrEP were more willing to have CLAI with a casual HIV-positive partner who has a UVL (48%) than HIV-negative and untested GBM not on PrEP (6%) [18]. Other studies have found PrEP use among GBM to be associated with higher rates of CLAI with HIV-positive partners compared to HIV-negative GBM not using PrEP [19, 20].

U = U is an ongoing global campaign [15], and there is evidence that belief in TasP is increasing among GBM [12]. Previous research has found PrEP-experienced GBM are more willing to have CLAI with a partner who has a UVL [18]. However, the proportion of those willing is not as high as would be expected, given the double protection provided by PrEP and a UVL. Therefore, a better understanding of this group is needed to reveal specific subgroups among PrEP-experienced GBM where more concentrated promotion may be required. Further, as knowledge of and belief in TasP continues to change over time, more recent data on this topic is needed. The aims of this study were to examine the willingness of PrEP-experienced GBM to have CLAI with an HIV-positive sexual partner who has a UVL, and to determine factors associated with willingness to have CLAI in the context of UVL among PrEP-experienced GBM.

## Methods

### Participants and Procedure

The *PrEP in NSW Transition Study* was a longitudinal study which enrolled previous participants of the *Expanded PrEP Implementation in Communities in New South Wales* (*EPIC-NSW*) study, a large-scale PrEP implementation trial in New South Wales (NSW), Australia. *EPIC-NSW* enrolled participants between March 2016 and April 2018, with follow-up until March 2019. In the original *EPIC-NSW* study, eligible participants were adults (aged ≥ 18 years) residing in NSW, who were at high risk of acquiring HIV as defined by the 2015 NSW Health PrEP guidelines [3, 21]. Detailed methodology has been published previously [3, 21]. At the conclusion of *EPIC-NSW*, participants who had completed optional, online, behavioural surveys on a quarterly basis during the study were invited via email to participate in the *PrEP in NSW Transition Study*. Recruitment was conducted between August 2018 and February 2019. Participants who consented to participate completed a survey through the online survey platform, SurveyGizmo, every 6 months from August 2018 to March 2020, completing three surveys in total. As participants were recruited from *EPIC-NSW*, all participants had experience taking PrEP, either previously or ongoing. The study received ethical approval from UNSW Sydney.

### Measures

Willingness to have CLAI with a HIV-positive partner who has a UVL was measured with the question, ‘I am willing to have anal sex without condoms with an HIV-positive partner who has an undetectable viral load’, with response options on a 6-point Likert scale ranging from 1 = ‘strongly disagree’ to 6 = ‘strongly agree’. Those who indicated agreement or strong agreement (scores 5–6) were classified as being ‘More willing to have CLAI with a HIV-positive partner who has a UVL’. Participants were asked a series of attitudinal items about HIV and STI prevention (with the same response options and dichotomised in the same way as above), including an item measuring their belief in the effectiveness of an undetectable viral load to prevent HIV transmission (‘An HIV-positive person having an undetectable viral load is effective at preventing HIV transmission’), hereafter referred to as ‘Belief in the effectiveness of TasP’.

Demographic items included age, gender, sexual identity, country of birth, postcode of residence, education, employment, and student status. Country of birth was categorised into six regions: Australia, high-income English-speaking countries (United Kingdom, Ireland, New Zealand, Canada, and United States), Europe, Asia, Latin America and the Caribbean, and other. Postcode of residence was categorised based on the percentage of gay-identified men residing in them, using a previously established method [22]. A previously established scale for measuring social engagement with other gay men (‘Gay Social Engagement’) with a range of 0–7 (7 indicating the highest GSE score), was calculated by adding numerically coded responses from two survey questions (number of gay friends, and free time spent with gay men) [23].

PrEP use was measured with items regarding current PrEP use (yes/no) and intended PrEP dosing regimen over the previous 6 months: daily, periodic (daily use during periods when participants believe themselves at high risk of acquiring HIV), event-driven (use around time of sexual encounter/s), time-based (use on specific days of the week), and other. PrEP use was classified as ‘current daily’, ‘current non-daily’, and ‘former user’. Adherence to PrEP was self-reported on a 0–100% scale, relative to the dosing regimen the participant reported. Participants were asked about their sexual behaviour in the past 6 months, including number of sexual partners and any group sex. Condomless intercourse in the past 6 months was measured by asking participants whether they engaged in CLAI and condomless vaginal intercourse with different types of sexual partners (HIV-positive with an undetectable viral load, HIV-positive with a detectable or unknown viral load, HIV-negative and taking PrEP, HIV-negative and not taking PrEP, and partners of unknown HIV status). Measures of insertive and receptive sexual positioning during intercourse were also derived from these items. Participants were also asked about their drug use in the past 6 months, including crystal methamphetamine, GHB, and erectile dysfunction medication, and whether they had used drugs for the purpose of sex.

### Statistical Analysis

Cross-sectional data from the most recent (12-month) survey, collected from October 2019 to March 2020, were used in this analysis, which was performed using Stata (version 17.0, StataCorp, College Station, Texas, USA). These data were used as they were the most recently collected. As the analysis focused on GBM (cisgender and transgender), female participants and heterosexual men were excluded from the analysis. To examine if our final cross-sectional sample differed from the baseline characteristics of the full *EPIC-NSW* cohort and those who completed the baseline survey of the *PrEP in NSW Transition Study*, we compared a number of demographic characteristics (age, gender, sexual identity, Indigenous status, and region of birth). Simple and multiple logistic regression models were conducted to determine associations with willingness to have CLAI with a HIV-positive partner who has a UVL. A sensitivity analysis was conducted by restricting the sample to participants who believed in the effectiveness of TasP, as willingness to have CLAI with a HIV-positive partner who has a UVL may have been confounded by lack of belief in the effectiveness of TasP. Odds ratios (OR), adjusted odds ratios (aOR), 95% confidence intervals (CI), and p-values were reported for the associations. Variables with a p-value of < 0.1 in the unadjusted models were block-entered into the multiple logistic regression models.

## Results

A total of 9596 participants were enrolled in *EPIC-NSW* and prescribed PrEP. Of those participants, 2344 completed the *PrEP in NSW Transition Study* baseline survey, and 1503 completed the 12-month follow-up survey. The final sample included in this analysis was 1386 GBM after excluding female participants (*n* = 6), participants who were not gay or bisexual (*n* = 2), and participants who had missing data (*n* = 109). The included sample was similar to the *EPIC-NSW* cohort and the baseline *PrEP in NSW Transition Survey* respondents (Table 1). However, mean age was higher in the included sample compared to the *EPIC-NSW* cohort and the baseline *PrEP in NSW Transition Survey* respondents, and there were slightly higher proportions of participants who identified as gay, and who were born in Australia (Table 1).

**Table 1:** Characteristics of the *EPIC-NSW* cohort, and *PrEP in NSW Transition Study* participants at baseline, and the included sample from the 12-month follow-up survey

	EPIC-NSW (N = 9596) *n* (%)	Baseline (N = 2344) *n* (%)	Included sample (N = 1386) *n* (%)
Age in years^a^	36.2 (10.8)	40.7 (11.4)	43.7 (11.6)
Gender			
Cis man	9455 (98.5)	2304 (98.3)	1371 (98.9)
Trans man	25 (0.3)	7 (0.3)	3 (0.2)
Non-binary	2 (0.3)	18 (0.8)	11 (0.8)
Other^b^	88 (0.10)	15 (0.6)	1 (0.07)
Sexual identity			
Gay sexual identity	8781 (91.5)	2183 (93.1)	1300 (93.8)
Other sexual identity	815 (8.5)	161 (6.9)	86 (6.2)
Aboriginal and/or Torres Strait Islander	129 (1.7)	45 (2.0)	20 (1.4)
Region of birth			
Australia	5059 (60.1)	1403 (60.8)	853 (61.5)
High-income English-speaking countries	1049 (12.5)	310 (13.4)	204 (14.7)
Asia	1240 (14.7)	298 (12.9)	160 (11.5)
Europe	370 (4.4)	106 (4.6)	68 (4.9)
Latin America and Caribbean	374 (4.4)	97 (4.2)	45 (3.3)
Other	326 (3.9)	92 (4.0)	56 (4.0)

^a^*Mean* (*SD*)

^b^Includes women, intersex people, and people who chose not to define their gender

In the final cross-sectional sample, mean age was 43.7 years (*SD* = 11.6), 98.9% of participants were cisgender men, 93.8% identified as gay, 1.4% were Aboriginal or Torres Strait Islander, and 61.5% were born in Australia. Nearly half of participants lived in a postcode in which fewer than 5% of resident men were estimated to identify as gay (*n* = 644, 46.5%), two-thirds were university educated (*n* = 937, 67.6%), three-quarters worked full-time (*n* = 1029, 74.0%), and 6.1% (*n* = 84) were full-time students. In the past 6 months, most participants had taken PrEP (*n* = 1127, 81.3%), and participants’ self-reported mean adherence to PrEP was 81.4% (*SD* = 33.3). Nearly half reported more than 10 sexual partners (*n* = 642, 46.3%), and 90.1% (*n* = 1249) reported having had condomless intercourse (anal and/or vaginal) in the previous 6 months. Of all participants, nearly half had condomless intercourse with an HIV-positive partner with a UVL (*n* = 560, 40.4%), 17.5% (*n* = 243) had condomless intercourse with a HIV-positive partner with a detectable or unknown viral load, 71.3% (*n* = 988) with a HIV-negative partner on PrEP, 66.7% (*n* = 924) with a HIV-negative partner not on PrEP, and 59.4% (*n* = 823) with a partner of unknown HIV status in the past 6 months. In the same period over half of participants had engaged in group sex at least once (*n* = 849, 61.3%), one-fifth had used crystal methamphetamine (*n* = 276, 19.9%), and nearly two-fifths used drugs for the purposes of sex (*n* = 477, 34.4%). Over three-quarters of participants believed in the effectiveness of TasP (*n* = 1095, 79.0%).

Over half of participants were classified as being willing to have CLAI with an HIV-positive partner who has a UVL (55.3%). Unadjusted models found that compared to men less willing to have CLAI with partner who has a UVL, those who were willing were more likely to: be older (*M* = 44.2 versus *M* = 43.1), identify as gay (95.2% versus 92.1%), have been born in Australia (64.1% versus 58.4%), live in a postcode where 5% or more residents identify as gay (58.2% versus 47.7%), and have higher gay social engagement (*M* = 4.71 versus *M* = 4.19; Table 2). They were also more likely to have, in the past 6 months, taken PrEP (86.3% versus 75.2%), had a higher adherence to PrEP (*M* = 85.2 versus *M* = 76.6), had more than 10 sexual partners (53.9% versus 36.9%), had condomless intercourse (92.0% versus 87.7%), been the insertive partner during intercourse (85.9% versus 78.2%), engaged in group sex (69.3% versus 51.3%), used crystal methamphetamine (27.2% versus 11.0%), used drugs for the purposes of sex (43.0% versus 23.9%), believe in the effectiveness of TasP (92.8% versus 61.9%), and prefer CLAI (over anal intercourse with a condom) (91.3% versus 68.4%; Table 2). Those who were willing to have CLAI with a partner who has a UVL were less likely to worry about getting HIV when taking PrEP (15.3% versus 27.4%), be concerned about getting STIs (67.0% versus 79.8%), and to try to avoid getting STIs (60.3% versus 73.9%).

**Table 2:** Variables associated with willingness to have CLAI with a HIV-positive partner with a UVL

	Less willing to have CLAI with UVL (*n* = 620) *n* (%)	More willing to to have CLAI with UVL (*n* = 766) *n* (%)		Unadjusted OR (95% CI)	*p*	aOR (95% CI)^a^		*p*	
Age^b^	43.1 (11.8)	44.2 (11.4)		1.01 (1.00–1.02)	0.086	1.01 (1.00–1.03)		**0.017**	
Cisgender man	615 (99.2)	756 (98.7)		0.61 (0.21–1.81)	0.377				
Gay sexual identity	571 (92.1)	729 (95.2)		1.69 (1.09–2.63)	0.020	1.17 (0.62–2.22)		0.631	
Australian-born	362 (58.4)	491 (64.1)		1.27 (1.02–1.58)	0.030	1.39 (1.07–1.81)		**0.013**	
≥ 5% gay-identified men in postcode	296 (47.7)	446 (58.2)		1.53 (1.23–1.89)	< 0.001	1.07 (0.82–1.39)		0.639	
University educated	421 (67.9)	516 (67.4)		0.98 (0.78–1.22)	0.831				
Employed full-time	448 (72.3)	581 (75.9)		1.21 (0.95–1.54)	0.129				
Full-time student	44 (7.2)	40 (5.2)		0.72 (0.46–1.12)	0.140				
Gay social engagement^b^	4.19 (1.54)	4.71 (1.39)		1.28 (1.18–1.37)	< 0.001	1.10 (1.00–1.20)		0.052	
PrEP use^c^	466 (75.2)	661 (86.3)		2.08 (1.58–2.74)	< 0.001	1.42 (0.82–2.45)		0.206	
Proportion of adherence to PrEP^b,c^	76.6 (36.8)	85.2 (29.7)		1.01 (1.00–1.11)	< 0.001	1.00 (0.99–1.01)		0.899	
More than 10 sexual partners^c^	229 (36.9)	413 (53.9)		2.00 (1.61–2.48)	< 0.001	1.56 (1.17–2.08)		**0.002**	
Condomless intercourse^c,d^	544 (87.7)	705 (92.0)		1.61 (1.13–2.30)	0.008	0.65 (0.40–1.08)		0.096	
Sexual positioning^c^									
Any insertive	485 (78.2)	658 (85.9)		1.70 (1.28–2.24)	< 0.001	1.22 (0.86–1.74)		0.267	
Any receptive	507 (81.8)	625 (81.6)		0.99 (0.75–1.30)	0.931				
Group sex^c^	318 (51.3)	531 (69.3)		2.15 (1.72–2.67)	< 0.001	1.23 (0.91–1.67)		0.180	
Crystal methamphetamine use^c^	68 (11.0)	208 (27.2)		3.03 (2.25–4.08)	< 0.001	2.41 (1.55–3.75)		**< 0.001**	
Drug use for purposes of sex^c^	148 (23.9)	329 (43.0)		2.40 (1.90–3.03)	< 0.001	1.15 (0.81–1.65)		0.434	
Attitudinal statements									
An HIV-positive person having an undetectable viral load is effective at preventing HIV transmission	384 (61.9)	711 (92.8)		7.94 (5.78–10.93)	< 0.001	7.60 (5.36–10.76)		**< 0.001**	
I prefer not to use condoms for anal sex	424 (68.4)	699 (91.3)		4.82 (3.56–6.53)	< 0.001	3.55 (2.51–5.02)		**< 0.001**	
When I am taking PrEP, I still worry about getting HIV	170 (27.4)	117 (15.3)		0.48 (0.37–0.62)	< 0.001	0.73 (0.53–0.99)		**0.046**	
I am concerned about getting STIs	495 (79.8)	513 (67.0)		0.51 (0.40–0.66)	< 0.001	0.57 (0.42–0.77)		**< 0.001**	
I try to avoid getting STIs	458 (73.9)	462 (60.3)		0.54 (0.43–0.68)	< 0.001	0.75 (0.57–1.00)		**0.048**	

Bold values indicate statistical significance

^a^Adjusted odds ratio

^b^*Mean* (SD)

^c^In last 6 months

^d^Includes both anal and vaginal intercourse

In multiple logistic regression, greater willingness to have CLAI with a HIV-positive partner who has a UVL was independently associated with being older (aOR 1.01; 95% CI 1.00–1.03), being born in Australia (aOR 1.39; 95% CI 1.07–1.81), having more than 10 sexual partners in the past 6 months (aOR 1.56; 95% CI 1.17–2.08), any crystal methamphetamine use in the past 6 months (aOR 2.41; 95% CI  1.55–3.75), believing in the effectiveness of TasP (aOR 7.60; 95% CI 5.36–10.76), and preference for CLAI (aOR 3.55; 95% CI 2.51–5.02; Table 2). Those who were willing to have CLAI with a partner who has a UVL were less likely to worry about getting HIV when taking PrEP (aOR 0.73; 95% CI 0.53–0.99), be concerned about getting STIs (aOR 0.57; 95% CI 0.42–0.77), or try to avoid getting STIs (aOR 0.75; 95% CI 0.57–1.00; Table 2).

When restricting the sample to the 1095 participants who believed in the effectiveness of TasP to conduct the sensitivity analysis, the results were predominantly the same as the original model (Supplementary Table 1). However, greater willingness to have CLAI with a HIV-positive partner who has a UVL was not associated with age in the simple logistic regression analysis, or with trying to avoid getting STIs in the multiple logistic regression analysis.

## Discussion

This study assessed the attitudes of PrEP-experienced GBM towards TasP and CLAI with virally suppressed HIV-positive partners. We found most men (79.0%) believed in the effectiveness of TasP. However, despite their belief in TasP, and that most participants were taking PrEP when surveyed, just over half (55.3%) were willing to have CLAI with a HIV-positive partner who has a UVL, and less than half (40.4%) reported having had condomless sexual intercourse (anal and/or vaginal) with a partner with a UVL. Those who were willing to have CLAI with a partner who has a UVL were less likely to worry about getting HIV when taking PrEP, and more likely to believe in the effectiveness of TasP.

Men who were willing to have CLAI with a HIV-positive partner who has a UVL were less likely to report worrying about getting HIV when taking PrEP. Those who worry may believe in the effectiveness of PrEP and TasP on an abstract level, but may nonetheless feel anxious about having CLAI with a HIV-positive partner who has a UVL due to fear of infection, despite potentially knowing their fears are not evidence-based [24]. Some men with anxiety about HIV transmission cite concerns about fluctuations in viral load, or generalised fear from being conditioned to consider HIV as a threat [24]. This may explain our finding that 35.1% of participants believed in the effectiveness of TasP but were not willing to have CLAI with a partner who has a UVL. This finding suggests that fear of HIV may potentially generate stigma towards people living with HIV, if those same participants are willing to have CLAI with HIV-negative partners.

Participants who were willing to have CLAI with an HIV-positive partner who has a UVL were less likely to be concerned about STIs, and more likely to try to avoid STIs. The gap between belief in TasP and willingness to have CLAI with a partner who has a UVL may therefore reflect that some participants who were unwilling to have CLAI with a partner with a UVL may not be concerned with the effectiveness of TasP, but rather, are concerned about the transmission of STIs through CLAI. Our results regarding willingness to have CLAI with a partner with a UVL may also be influenced by other factors. For instance, some participants could have indicated that they would be unwilling to have CLAI with a partner with a UVL because they dislike anal intercourse, rather than due to a distrust of TasP or stigma towards HIV-positive partners. Such participants would therefore be unwilling to have CLAI with a partner of any HIV status, but may be willing to engage in other types of sexual behaviour with a partner with a UVL.

Consistent with previous research [14, 25], men who were willing to have CLAI with a partner who has a UVL were more likely to believe TasP is effective at preventing HIV transmission. Having greater knowledge of TasP and personal experience using it has been reported to increase belief in the ability of TasP to prevent HIV transmission [25, 26]. This reduces fear of transmission and increases the extent to which those who believe in TasP are willing have CLAI with a partner who has a UVL [25, 26]. Therefore, participants in the current study who believed in the effectiveness of TasP may have had more knowledge of TasP, and/or experience using it, compared to those who did not believe in it.

Men who were willing to have CLAI with a HIV-positive partner who has a UVL were more likely to be current PrEP users, and to have a higher adherence to PrEP (although these associations were not statistically independent after adjusting for other variables). This may be because PrEP enables current users to feel more comfortable having CLAI than former PrEP users or those with lower adherence to PrEP. Qualitative research has found some PrEP-experienced GBM use PrEP as a supplementary layer of protection against potential HIV exposure when using other prevention strategies, such as condoms [27].

Participants who were willing to have CLAI with a HIV-positive partner who has a UVL were more likely to be born in Australia. Previous research has found that compared to gay male HIV serodiscordant couples in Brazil and Thailand, Australian couples relied more heavily on TasP [28]. Previous research has found a lack of knowledge of and confidence in TasP among overseas-born men in Australia and Canada [29, 30], and that understanding of HIV prevention extends primarily to condoms among many overseas-born GBM in Australia [31]. Given willingness to have CLAI with a partner who has a UVL typically increases with knowledge of TasP [25], this finding may reflect a lack of knowledge regarding TasP among overseas-born GBM. This may be because in some countries, HIV is highly stigmatised, and consequently there is less community dialogue and awareness of TasP, and HIV more broadly [28, 31]. Some healthcare providers who are themselves sceptical of the effectiveness of TasP are also unwilling to discuss it with their patients [32].

Participants who were willing to have CLAI with a HIV-positive partner who has a UVL were more likely to have a higher number of sexual partners, and to report using crystal methamphetamine. Our findings align with previous research which has found that more ‘sexually adventurous’ or highly sexually active GBM are more likely to use HIV prevention and risk reduction strategies [33, 34]. These GBM may be more willing to have CLAI with a partner who has a UVL because they are already engaging in CLAI and/or using risk reduction strategies that enable them to have CLAI without worrying about HIV transmission. Therefore, TasP may provide such GBM with a layer of protection against the CLAI they were already engaging in. This is particularly likely given CLAI was a prerequisite for participants’ enrolment and ongoing eligibility in *EPIC-NSW* [21].

Participants who were more willing to rely on TasP were more likely to have a high level of gay social engagement (although this association was not statistically independent after adjusting for other variables). Those with strong gay community networks may be more likely to see HIV prevention campaign material, which is promoted in gay media, and at gay venues and events [35]. Further, those in gay community networks spread HIV prevention information among their networks, and normalise novel HIV prevention strategies [36]. Exposure to HIV prevention information would likely increase GBM’s knowledge of TasP [25], and thereby increase their belief in TasP and willingness to have CLAI with a HIV-positive partner who has a UVL.

Several implications for further research, policy, and practice arise from our findings. First, further research is needed to understand the gap between belief in TasP and willingness to have CLAI with a HIV-positive partner who has a UVL. This would enable subsequent creation of government and community-based responses to address this gap. In particular, understanding the specific role of HIV stigma in the gap between belief in TasP and willingness to have CLAI with a partner who has a UVL would be beneficial. Future studies on sexual behaviour and HIV risk among GBM could be improved by incorporating items regarding HIV stigma. HIV stigma is also a key priority for policymakers globally [37] and in Australia [38, 39]. Further efforts are needed in policy and health promotion to address HIV stigma. Second, our findings suggest more work is needed to promote TasP to overseas-born GBM living in Australia, and to address barriers they may face to accessing HIV prevention information. Since the data for this study was collected, HIV prevention campaigns in Australia have been executed in several community languages [35] and there is an increasing focus on migrants in research and health promotion, indicating work to address such barriers is underway in Australia. Finally, clinicians could also play a role by promoting HIV prevention strategies such as TasP to their patients, and reduce stigma and anxiety by addressing any misconceptions or concerns their patients have in relation to the effectiveness of an undetectable viral load to prevent HIV transmission.

There are limitations in our study which should be considered. This study used an online, volunteer sample from the *EPIC-NSW* study, a PrEP implementation trial. Participants who volunteered for the study were ‘early adopters’ of PrEP in Australia and may have been more willing to experiment with other biomedical HIV prevention strategies, such as TasP [18]. The study used self-reported data, and so may have been impacted by recall error and social desirability bias. However, participants were informed that the survey was anonymous, which may have mitigated this. Participants had a mean age of 43 years, the majority were university educated, employed full time, and had higher gay social engagement. This may limit generalisability to younger GBM, and GBM who are not well connected to other gay men. The results of this study may also not be generalisable to PrEP-naïve GBM. Highly sexually active GBM have been found to be more likely to initiate PrEP [40], and GBM using PrEP are more likely to use TasP and engage in CLAI compared to HIV-negative GBM not using PrEP [18, 19]. This study was not specifically designed to examine HIV stigma, and therefore there were no items regarding HIV stigma in the survey. The study also only examined participants’ willingness to have CLAI with partners with a UVL, and therefore the results cannot be extrapolated to other sexual behaviours or partner types. Finally, as this was a cross-sectional study, we cannot infer causal relationships between the variables associated with willingness to have CLAI with a partner who has a UVL.

## Conclusion

While most PrEP-experienced GBM in this study believed in the effectiveness of TasP, only half were willing to have CLAI with a HIV-positive partner who has a UVL. Those who were willing to have CLAI with a partner who has a UVL were less likely to worry about getting HIV when taking PrEP, and were more likely to believe in the effectiveness of TasP. PrEP-experienced GBM who believe in the effectiveness of TasP, but are less willing to have CLAI with a partner who has a UVL may still fear HIV transmission despite their belief in the effectiveness of TasP. Further work is needed to understand the gap between belief in TasP and willingness to have CLAI with a partner who has a UVL among PrEP-experienced GBM, and the role caution, fear and HIV stigma may play in these attitudes.

## Supplementary Information

Below is the link to the electronic supplementary material.

Supplementary file1 (DOCX 21 kb)

## Data Availability

Not applicable.
