# Supplementary material for: Attitudes Towards Treatment as Prevention Among PrEP-Experienced Gay and Bisexual Men in Australia
Source: AIDS Behav. 2023 Mar 6;27(9):2969–78. doi: 10.1007/s10461-023-04019-x (PMC10386911; doi:10.1007/s10461-023-04019-x)
Supplement: Supplementary file 1 — Supplementary file1 (DOCX 21 kb) [file 10461_2023_4019_MOESM1_ESM.docx]

| **Supplementary Table I**: Variables associated with willingness to have CLAI with a HIV-positive partner with a UVL, restricted to men who believed TasP prevents HIV transmission. | | | | | | |
| --- | --- | --- | --- | --- | --- | --- |
|  | **Less willing to have CLAI with UVL**  **(*n*=384)**  ***n* (%)** | **More willing to have CLAI with UVL**  **(*n*=711)**  ***n* (%)** | **Unadjusted OR (95% CI)** | ***p*** | **aOR (95% CI)^a^** | ***p*** |
| Age^b^ | 42.8 (11.2) | 43.9 (11.4) | 1.01 (1.00-1.02) | 0.126 |  |  |
| Cisgender man | 379 (98.7) | 701 (98.6) | 0.92 (0.31-2.73) | 0.887 |  |  |
| Gay sexual identity | 357 (93.0) | 679 (95.5) | 1.60 (0.95-2.72) | 0.079 | 1.21 (0.62-2.38) | 0.576 |
| Australian-born | 212 (55.2) | 456 (64.1) | 1.45 (1.13-1.87) | 0.004 | 1.44 (1.09-1.91) | **0.011** |
| ≥ 5% gay-identified men in postcode | 198 (51.6) | 418 (58.8) | 1.34 (1.04-1.72) | 0.022 | 1.05 (0.78-1.39) | 0.763 |
| University educated | 277 (72.1) | 486 (68.4) | 0.83 (0.63-1.10) | 0.194 |  |  |
| Employed full-time | 280 (72.9) | 539 (75.8) | 1.16 (0.88-1.54) | 0.293 |  |  |
| Full-time student | 31 (8.1) | 36 (5.1) | 0.61 (0.37-1.00) | 0.048 | 0.95 (0.53-1.69) | 0.855 |
| Gay Social Engagement^b^ | 4.35 (1.49) | 4.74 (1.38) | 1.21 (1.11-1.32) | <0.001 | 1.10 (1.00-1.22) | 0.055 |
| PrEP use^c^ | 295 (76.8) | 615 (86.5) | 1.93 (1.40-2.66) | <0.001 | 1.50 (0.85-2.65) | 0.157 |
| Proportion of adherence to PrEP^b,c^ | 78.0 (35.5) | 85.3 (29.3) | 1.01 (1.00-1.01) | <0.001 | 1.00 (0.99-1.01) | 0.989 |
| More than 10 sexual partners^c^ | 141 (36.7) | 383 (53.9) | 2.01 (1.56-2.60) | <0.001 | 1.52 (1.12-2.08) | **0.008** |
| Condomless intercourse^c,d^ | 338 (88.0) | 658 (92.6) | 1.69 (1.11-2.56) | 0.014 | 0.73 (0.42-1.26) | 0.252 |
| Sexual positioning^c^ |  |  |  |  |  |  |
| Any insertive | 306 (79.7) | 614 (86.4) | 1.61 (1.16-2.24) | 0.004 | 1.19 (0.80-1.76) | 0.386 |
| Any receptive | 316 (82.3) | 582 (81.9) | 0.97 (0.70-1.34) | 0.858 |  |  |
| Group sex^c^ | 196 (51.0) | 497 (69.9) | 2.23 (1.72-2.88) | <0.001 | 1.30 (0.93-1.79) | 0.120 |
| Crystal methamphetamine use^c^ | 39 (10.2) | 191 (26.9) | 3.25 (2.24-04.71) | <0.001 | 2.62 (1.59-4.32) | **<0.001** |
| Drug use for purposes of sex^c^ | 96 (25.0) | 304 (42.8) | 2.24 (1.70-2.95) | <0.001 | 1.04 (0.70-1.55) | 0.831 |
| Attitudinal Statements |  |  |  |  |  |  |
| I prefer not to use condoms for anal sex | 271 (70.6) | 649 (91.3) | 4.36 (3.10-6.14) | <0.001 | 3.32 (2.28-4.85) | **<0.001** |
| When I am taking PrEP, I still worry about getting HIV | 91 (23.7) | 105 (14.8) | 0.56 (0.41-0.76) | <0.001 | 0.67 (0.47-0.94) | **0.021** |
| I am concerned about getting STIs | 306 (79.7) | 477 (67.1) | 0.52 (0.39-0.70) | <0.001 | 0.58 (0.42-0.81) | **0.001** |
| I try to avoid getting STIs | 284 (74.0) | 434 (61.0) | 0.55 (0.42-0.73) | <0.001 | 0.82 (0.60-1.11) | 0.199 |
| Bold values indicate statistical significance  ^a^Adjusted odds ratio  ^b^*Mean* (SD) |  |  |  |  |  |  |
| ^c^In last 6 months  ^d^Includes both anal intercourse and penetrative (vaginal) intercourse | | | | | | |
